# The Association between Grazing and Food Addiction: The Italian Version of the Repetitive Eating Questionnaire (Rep(Eat)-Q) and Its Relationships with Food Addiction Criteria

**DOI:** 10.3390/nu16070949

**Published:** 2024-03-26

**Authors:** Alessandro Alberto Rossi, Stefania Mannarini, Michelle Semonella, Gianluca Castelnuovo, Giada Pietrabissa

**Affiliations:** 1 Department of Philosophy, Sociology, Education, and Applied Psychology, Section of Applied Psychology, University of Padova, 35131 Padova, Italy; stefania.mannarini@unipd.it; 2 Interdepartmental Center for Family Research, University of Padova, 35131 Padova, Italy; 3 Department of Psychology, Bar-Ilan University, Ramat Gan 5290002, Israel; michelle.semonella@biu.ac.il; 4 Department of Psychology, Catholic University of Milan, 20123 Milan, Italy; gianluca.castelnuovo@unicatt.it (G.C.); giada.pietrabissa@unicatt.it (G.P.); 5 Clinical Psychology Research Laboratory, IRCCS Istituto Auxologico Italiano, 20149 Milan, Italy

**Keywords:** grazing, food addiction, repetitive eating, eating compulsivity, compulsive grazing, compulsive eating, emotional eating, obesity

## Abstract

Background: Among the dysfunctional eating behaviors associated with excessive food intake, a construct that is gaining increasing attention is grazing—the constant, continuous, compulsive, and repetitive consumption of small/moderate amounts of food. Furthermore, in some cases, grazing seems to indicate a dependence on food and/or eating. Currently, the Repetitive Eating Questionnaire (Rep(Eat)-Q) appears to be the only questionnaire that comprehensively measures grazing, including its repetitive and compulsive eating component. Therefore, in a sample of individuals with severe obesity, the objective of this study was twofold: (A) to evaluate the psychometric properties of the Italian version of the Rep(Eat)-Q, and (B) to analyze the association between grazing and food addiction (FA). Method: A cross-sectional research design was used. A total of 402 inpatients with severe obesity (BMI > 35) were recruited. Participants underwent a series of questionnaires to investigate structural validity and convergent validity and association with FA criteria. Results: The factorial structure of the Rep(Eat)-Q is robust and showed fit indexes: CFI = 0.973; RMSEA = 0.074; 90%CI [0.056–0.091]; and SRMR = 0.029. Also, it exhibited good internal consistency and convergent validity. Furthermore, logistic regression analysis highlights a specific association between certain FA criteria and grazing. Conclusions: The Rep(Eat)-Q can be considered to be a concise, robust, reliable, and statistically sound tool to assess repetitive eating, specifically grazing. Its strong psychometric properties offer significant advantages for both research and clinical applications. Furthermore, in a sample of individuals with severe obesity, the results suggest that individuals with problematic grazing exhibit a typical behavioral profile of subjects with FA, indicating that FA can manifest through problematic grazing as well.

## 1. Introduction

The prevalence of people with obesity worldwide continues to increase, particularly in industrialized countries, where it has been projected that more than 85% of adults will be affected by overweight or obesity in the coming years [1,2,3]. Consequently, healthcare costs associated with overweight and obesity account for almost 10% of national healthcare expenditure [4], and it is anticipated that these costs will continue to increase over the next 15 years [5,6].

Obesity is a multifactorial chronic disease influenced by various biological factors (e.g., genetic, medical), psychological factors, and situational factors that contribute to its development and persistence [1,5,7,8,9]. One potentially crucial aspect implicated in the development and persistence of obesity is the idea that certain foods may trigger a dependency response in some individuals [10,11,12]—namely, food addiction (FA). FA is a complex concept that involves compulsive overeating and a loss of control over food consumption, characterized by behaviors similar to those seen in substance use disorders (SUDs) [13,14,15,16,17].

It has, therefore, a dual nature [18]: a component associated with SUDs and a component related to the nature of eating disorders (EDs) [19,20,21,22,23,24].

Specifically, being driven by unhealthy eating patterns [25,26,27,28,29,30], the ease of access to highly processed foods (HPFs) [31,32,33]—which are highly palatable and psychologically rewarding [34,35,36,37,38]—may predispose people to develop an addiction. These foods have the potential to activate neural reward systems [34,35,36,37,38], requiring the individual to seek the same feelings of well-being and pleasure. If these cravings are not satisfied, this could lead to the appearance of withdrawal symptoms [39,40,41]. Furthermore, the sustained consumption of HPFs can lead the individual to develop tolerance to the substance, thus necessitating increased intake to achieve the same rewarding effect.

On the other hand, FA can promote dysfunctional eating behaviors, such as spending a significant amount of time thinking about food or, as observed in cases of emotional eating, using food as an external regulator for intense (often negative) and/or uncontrollable emotions [42,43,44,45]. Additionally, individuals with FA commonly experience significant social and psychological impairment [24,46] attributed to cravings for HPFs [35] and an inability to stop or moderate their intake, even when they recognize negative consequences [16,47]. Furthermore, when attempting to reduce or eliminate the consumption of addictive foods, people can experience withdrawal symptoms such as irritability, anxiety, or mood swings [48]. This could lead to the misconception that binge eating behavior could represent a prototypical manifestation of FA [49,50,51,52]. However, while some individuals who engage in binge eating may exhibit behaviors similar to those seen in substance addiction, such as engaging in episodes of compulsive overeating characterized by a loss of control and consuming large amounts of food in a short period of time, not all instances of binge eating can be attributed to FA.

Binge eating disorder (BED), for example, is a distinct psychological condition characterized by recurrent episodes of uncontrollably eating large amounts of food in a short period, often to the point of discomfort or distress. Individuals with BED may experience feelings of guilt, shame, or a loss of control during or after binge eating episodes. While there may be overlap in some symptoms with addictive behaviors, such as cravings and food preoccupation, BED is recognized as a separate disorder in the diagnostic and statistical manual of mental disorders (DSM-5). Notably, FA is instead not yet formally recognized as a diagnostic category in the DSM-5.

In addition, food addiction-like behaviors, such as eating large quantities of food followed by purging or compensatory behaviors, can be present in individuals with bulimia nervosa (BN), and, although less common, some individuals with anorexia nervosa (AN) may also experience FA during periods of binge eating or when attempting to reintroduce food after prolonged restriction.

Recent research findings also indicates an association between FA and other problematic eating patterns [53,54], such as grazing behavior [55,56,57]. It is defined as the constant, repetitive, compulsive, and unplanned consumption of small/moderate amounts of food throughout the day, commonly without structured meal times [55,58,59]. According to the conceptualization provided by Conceição and colleagues [58], there are two types of grazing. The first type of grazing (non-compulsive grazing/repetitive eating) represents a manner of eating distractedly and without particular thoughts [55,58]. This type of grazing is associated with a lesser loss of control and is also less predisposing to binge eating behavior [60]. The second type (compulsive grazing) would reflect the feeling that the individual cannot resist food and that the individual is tempted to eat again even when trying to resist [55,58]. This type of grazing is associated with a larger loss of control, predisposing to binge eating behavior, and subsequent negative emotional states. Compulsive grazing appears to be associated with greater psychological distress [55], including negative affect [61], anxiety, depression, and poorer mental health [49]. Furthermore, it is associated with a higher body mass index (BMI), food inhibition, and hunger [49], as well as the failure to lose weight after bariatric surgery interventions [49,60,62,63]. In this regard, evidence suggests that patients who exhibit binge eating behaviors before surgery might develop grazing behaviors after surgery, especially when large quantities of food cannot be consumed [49,64].

While not classified as a distinct diagnosis in the DSM-5, grazing behavior warrants attention in the assessment and treatment of eating disorders, particularly BED and other specified feeding or eating disorders, as it may also be observed in individuals with atypical presentations of eating disorders.

For the systematic screening and early identification of grazing behavior [55,58], Conceição and colleagues (2017) developed a brief, easily administered self-report tool: the Repetitive Eating Questionnaire (Rep(Eat)-Q) [55]. It comprises 12 easily interpretable items measured on a Likert scale, which refer to two distinct but highly correlated factors: the compulsivity factor and the repetitiveness factor. In the original validation study [55], the questionnaire was administered to two different populations: a community sample and a clinical sample of individuals enrolled in a bariatric surgery program.

However, despite its importance as a predictive factor for weight loss, to date the Rep(Eat)-Q is not available for use in the Italian population [62], and the relationship between grazing and FA requires further empirical confirmation. Therefore, considering a sample of individuals with severe obesity, the purpose of this study was twofold: (A) to evaluate the psychometric properties of the Italian version of the Rep(Eat)-Q (Part I) and (B) to shed new light on the association between grazing and FA (Part II).

## 2. Materials and Methods

### 2.1. Translation and Cultural Adaptation

The Rep(Eat)-Q underwent a process of translation and cultural adaptation conforming to international guidelines [65]. Thus, to ensure consistency across languages, a back-to-back translation process was carried out. The final translation (see Supplementary Material) was then administered to a sample of 10 individuals to assess the comprehensibility of the items. No further adjustments were needed. The Italian version of the Rep(Eat)-Q is reported in the Supplementary Materials.

### 2.2. Sample Size Determination

The decision regarding the sample size was made a priori. According to guidelines [66,67], the ‘*n:q*’ criterion (that is, the number of individuals per parameter) was adopted. A minimum sample of 10 individuals per parameter (=25) was enrolled. Consequently, a minimum sample of 250 participants was ensured.

### 2.3. Procedure

According to previous studies [68,69], participants were individually enrolled in the IRCCS Istituto Auxologico Italiano, San Giuseppe Hospital in Piancavallo, Verbania, Italy—a hospital for the treatment and rehabilitation of people with severe obesity (BMI > 35). The recruitment materials provided comprehensive information about eligibility criteria and further details to ensure that participants could make informed decisions. This included guarantees of anonymity of responses.

Inclusion criteria for participating in the study were as follows: (A) native-Italian speaker, (B) aged ≥18 years, and (C) having a BMI > 35. On the contrary, subjects were excluded from the study if (A) they were unable to complete the survey and (B) they did not provide informed consent.

This study received approval from the Ethics Committee of the IRCCS Istituto Auxologico Italiano (protocol no 2020_02_18_04).

### 2.4. Participants

A sample of 402 participants with severe obesity was enrolled. The sample was composed of 179 (44.5%) males and 223 (55.5%) females, aged 19 to 82 (*mean* = 55.25, *SD* = 12.92), with a body mass index (BMI) that ranged from 35.08 to 79.27 (*mean* = 42.28, *SD* = 6.46).

### 2.5. Measures

Respondents were asked to explain their main socio-demographic (i.e., age, sex, civil and educational status) and clinical characteristics (i.e., height and weight to compute the BMI).

#### 2.5.1. The Repetitive Eating Questionnaire (Rep(Eat)-Q)

The Rep(Eat)-Q [55] is a brief self-report measure developed to assess the frequency of attitudinal and behavioral features of grazing over the past 28 days (see Supplementary Materials). It comprises 12 items answered on a 7-point Likert-type scale ranging from 0 (Never) to 6 (Every day), with higher scores representative of higher frequency. The Rep(Eat)-Q is composed of two scales. The first, repetitive eating (RE), measures grazing associated with eating in a distracted, disorderly, and unaware manner, which seems to be predisposed to binge eating. The second scale, compulsive grazing (CG), measures the behavior of taking small/moderate amounts of food through involuntary and compelling behaviors—which the individual cannot resist—and that can cause distress. The total score and two scales, CG and RE, are calculated by averaging the scale items. A cut-off score of 1.25 on the total score suggests the presence of problematic/pathological grazing. The Rep(Eat)-Q is currently the grazing measure with the strongest psychometric support in the literature [70].

#### 2.5.2. The Modified Yale Food Addiction Scale 2.0 (mYFAS 2.0)

The mYFAS 2.0 [71,72] is a self-report tool designed to evaluate addictive eating behaviors through 13 items, each rated on an 8-point Likert-type scale. Of these items, 11 correspond to the DSM-5 diagnostic criteria for substance use disorder (SUD), while the other 2 specifically address the food-related deterioration or emotional distress experienced by the individual during the preceding 12 months. To diagnose FA, two scoring procedures must be considered: the symptom count score, which counts the diagnostic criteria met by the individual, and the diagnostic score, which considers the presence of impairment/emotional distress criteria (No FA; mild FA; moderate FA; and severe FA) [71]. In the current study, the mYFAS 2.0 shows a McDonald’s omega for categorical data equal to 0.908.

#### 2.5.3. The Binge Eating Scale (BES)

The BES [73,74] is used as a self-report instrument to measure the intensity of binge eating in various settings, including both community and clinical ones [24,75]. It comprises 16 questions that describe both behavioral aspects of BED, such as fast food or consuming large quantities of food, and associated feelings and cognitions, such as the fear of not being unable to stop eating. Each item has three to four levels of symptom descriptions. For BED to be diagnosed, a cut-off point should be reached in the total score [76]. The reliability and validity of the BES as a measure of eating-related pathology are confirmed in several studies with both clinical and community samples [77,78]. The internal consistency (McDonald’s omega) of the BES in the present study was 0.909.

#### 2.5.4. The Measure of Eating Compulsivity (MEC10)

The MEC10 [54,79] is a brief, feasible, solid, and extremely reliable tool consisting of 10 items answered on a 5-point Likert-type scale aimed at measuring compulsive eating behaviors and binge eating behaviors. High scores correspond to a high degree of eating compulsivity. A cut-off score suggests the presence of BED. The internal consistency (McDonald’s omega) of the MEC10-IT in the present study was equal to 0.949.

#### 2.5.5. The Three-Factor Eating Questionnaire Revised—18 (TFEQ-R-18)

The TFEQ-R-18 [80,81] is a reliable, solid, and psychometrically sound questionnaire that consists of 18 items measured on a 4-point Likert-type scale designed to assess three main cognitive and behavioral domains of eating disorders: cognitive restraint (CR), uncontrolled eating (UE), and emotional eating (EE). High scores reflect a higher level of each dimension. In this study, the internal consistency (McDonald’s omega) of the IT-TFEQ-R-18 scales was 0.747 for the CR scale, 0.914 for the UE scale, and 0.874 for the EE scale.

### 2.6. Statistical Analysis

Statistical analyses were run with R software (v. 4.3.2) and the following packages: ggplot2 [82], lavaan [83], lme4 [84], psych and psychTools [85,86], semPlot [87], and tidyverse [88].

**Figure 1:**
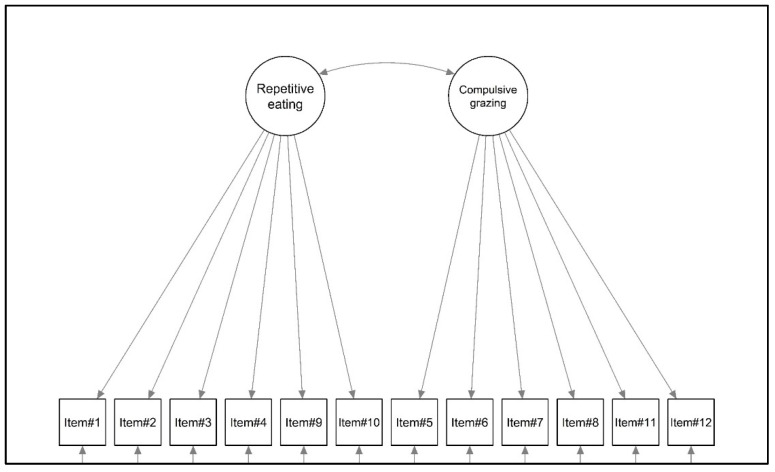
Graphical representation of the factorial structure of the Rep(Eat)-Q.

Considering the *first objective of this study*, a confirmatory factor analysis (CFA) was performed. According to the original validation study [55], a first-order model with a correlated factor was specified (see Figure 1).

Taking into account that some items should not be normally distributed, the MLR estimator (that is, robust maximum likelihood) was employed to evaluate the factorial structure of the Rep(Eat)-Q [66,67]. The fit was assessed using (A) the Yuan–Bentler chi-square statistic (YBχ^2^), (B) the approximation error of approximation (RMSEA), (C) the comparative fit index (CFI), and (D) the Standardized Root Mean Residual (SRMR). To assess the goodness of fit, cutoff criteria were used: (A) statistical nonsignificance of YBχ^2^, (B) an RMSEA lower than 0.08, (C) a CFI higher than 0.95, and (D) an SRMR lower than 0.08 [67,89,90].

Once the factorial structure of the Rep(Eat)-Q was tested, its internal consistency was evaluated with McDonald’s omega (ω) [91]. Additionally, the adjusted item-total correlation was calculated [92]. The assessment of convergence validity was performed using the Pearson correlation coefficient [92], with interpretations guided by Cohen’s benchmarks: *r* < 0.10, negligible; r ranging from 0.10 to 0.30, minimal; r ranging from 0.30 to 0.50, moderate; and *r* > 0.50, large [93].

Furthermore, considering the potential difference between males and females, a series of pairwise comparisons (independent sample *t*-tests) were conducted: independent variable, sex; dependent variables, Rep(Eat)-Q total score, Rep(Eat)-Q RE, and Rep(Eat)-Q CG. The results were interpreted using Cohen’s *d* and its benchmarks: small (*d*: 0.20 to 0.49), moderate (*d*: 0.50 to 0.79), and large (*d* > 0.80) [93].

Taking into account the *second aim of this study*, to test the association between problematic grazing and FA criteria, two different statistical analyses were performed. In each of them, grazing was used as a dichotomous dependent variable by dividing participants into two groups (0 = *non*-problematic grazing vs. 1 = problematic grazing) using the cut-off of the Rep(Eat)-Q (=1.25); meanwhile, FA criteria—measured with the mYFAS2.0 (0 = *non*-endorsed vs. 1 = endorsed)—were used as independent variable(s).

First, a series of simple bivariate chi-square tests (χ^2^) (2 × 2 contingency tables) were performed to test the simple bivariate association between the criteria of grazing and FA. The strength of the association was measured with the *Phi* (ϕ) coefficient, which was interpreted with Cohen’s benchmarks [93]: ϕ < 0.10, negligible; ϕ ranging from 0.10 to 0.30, minimal; ϕ from 0.30 to 0.50, moderate; and ϕ > 0.50, large. Furthermore, for each simple bivariate association, the odds ratio was also calculated: a positive odds ratio (OR) suggests that as the approval of FA criteria (independent variable) increases, there is a higher likelihood of problematic grazing (outcome).

Second, to verify the actual contribution of each FA criterion (controlling for all FA criteria) to the probability of presenting problematic grazing, a multiple logistic regression analysis was performed. The Hosmer–Lemeshow’s test was performed to test the goodness of model fit (a non-significant *p*-value is preferred). Cox and Snell’s PseudoR^2^ and Nagelkerke’s PseudoR^2^ coefficients were chosen as indices of the degree of variance explained. Furthermore, the OR for each predictor was calculated; even in this case, a positive odds ratio indicates that an increase in the acceptance of FA criteria (independent variable) is associated with a higher probability of experiencing problematic grazing (outcome). All regression coefficients (β) were not standardized.

## 3. Results

### 3.1. Part I: Psychometric Properties of the Italian Rep(Eat)-Q

#### 3.1.1. Structural Validity

The two-factor model (Figure 1) showed a good fit to the data for the sample of patients with severe obesity; all of the fit indices revealed a good fit to the data: YBχ^2^ (53) = 121.750, *p* < 0.001, the CFI = 0.973, RMSEA = 0.074, 90%CI [0.056–0.091], *p* (RMSEA < 0.05) < 001, and SRMR = 0.029. The standardized covariance between latent factors was equal to 0.926. Standardized factor loadings ranged from 0.689 (item#1—Repetitive eating) to 0.899 (item#10—Repetitive eating). All statistics are shown in Table 1.

#### 3.1.2. Internal Consistency

Internal consistency analysis shows excellent values for the two repeat scales (RE McDonald’s omega = 0.938; CG McDonald’s omega = 0.927) and for all aggregate items (McDonald’s omega = 0.960).

#### 3.1.3. Convergent Validity

As shown in Table 2, large correlations were found among the Rep(Eat)-Q scales and the total score. It should be noted that the Rep(Eat)-Q scales exhibit high associations with convergent validity scales that are linked to excessive food intake and uncontrolled behaviors.

Considering the Rep(Eat)-Q total score, a large association was found with the TFEQ-R-18 UE scale (*r* = 0.753, *p* < 0.001), the BES (*r* = 0.694, *p* < 0.001), and the mYFAS2.0 symptom count (*r* = 0.694, *p* < 0.001). Moreover, considering the Rep(Eat)-Q repetitive eating scale, a large association was found with the TFEQ-R-18 UE scale (*r* = 0.690, *p* < 0.001), the mYFAS2.0 symptom count (*r* = 0.639, *p* < 0.001), and the BES (*r* = 0.633, *p* < 0.001). Lastly, considering the Rep(Eat)-Q compulsive grazing scale, a large association was found with the TFEQ-R-18 UE scale (*r* = 0.762, *p* < 0.001), the MEC10 (*r* = 0.737, *p* < 0.001), and the BES (*r* = 0.706, *p* < 0.001)

#### 3.1.4. Differences between Males and Females

**Figure 2:**
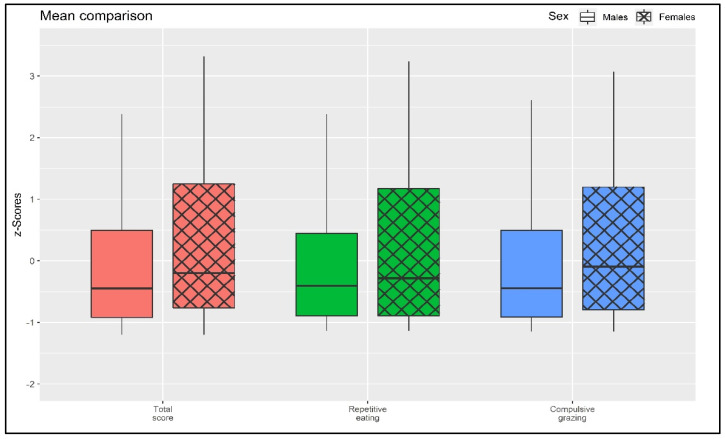
Mean comparison.

As shown in Figure 2, small-to-moderate differences were found between males and females, with the female sample scoring higher on all scales. Specifically, considering the total grazing scale (Rep(Eat)-Q total), males (*M* = 1.404; *SD* = 1.310) reported slightly lower scores compared to females (*M* = 1.962; *SD* = 1.652): *t* = −3.685; *p* < 0.001; *d* = |0.370|. Furthermore, considering the RE scale, males (*M* = 1.417; *SD* = 1.373) reported slightly lower scores compared to females (*M* = 1.814; *SD* = 1.675): *t* = −2.559; *p* = 0.011; *d* = |0.275|. Lastly, also considering the CG scale, males (*M* = 1.392; *SD* = 1.367) reported slightly lower scores compared to females (*M* = 2.110; *SD* = 1.725): *t* = −4.540; *p* < 0.001; *d* = |0.456|.

### 3.2. Part II: Association between Grazing and Food Addiction Criteria

#### 3.2.1. Bivariate Associations

**Figure 3:**
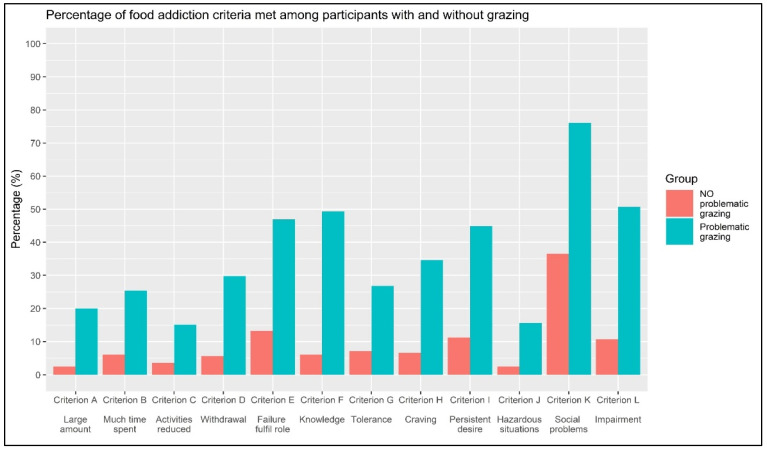
Percentage of food addiction criteria met among participants with and without problematic grazing.

A series of chi-square tests were conducted to assess the association between grazing (non-problematic vs. problematic) and meeting the criteria for FA. As reported in Table 3 and Figure 3, all criteria for FA are statistically significantly associated (all *p*-values < 0.001) with grazing, with an effect size ranging from small (ϕ = 0.198; Criterion C, “*Social activities given up or reduced*”) to moderate/high (ϕ = 0.480; Criterion F, “*Use despite knowledge of adverse consequences*”). Furthermore, a simple bivariate OR indicates that endorsing Criterion C for FA is associated with a 4.863 times higher risk of having problematic grazing compared to those who do not endorse it. Also, endorsing Criterion F for FA is associated with a 15.972 times higher risk of having problematic grazing compared to those who do not endorse it.

#### 3.2.2. Logistic Regression Analysis

**Figure 4:**
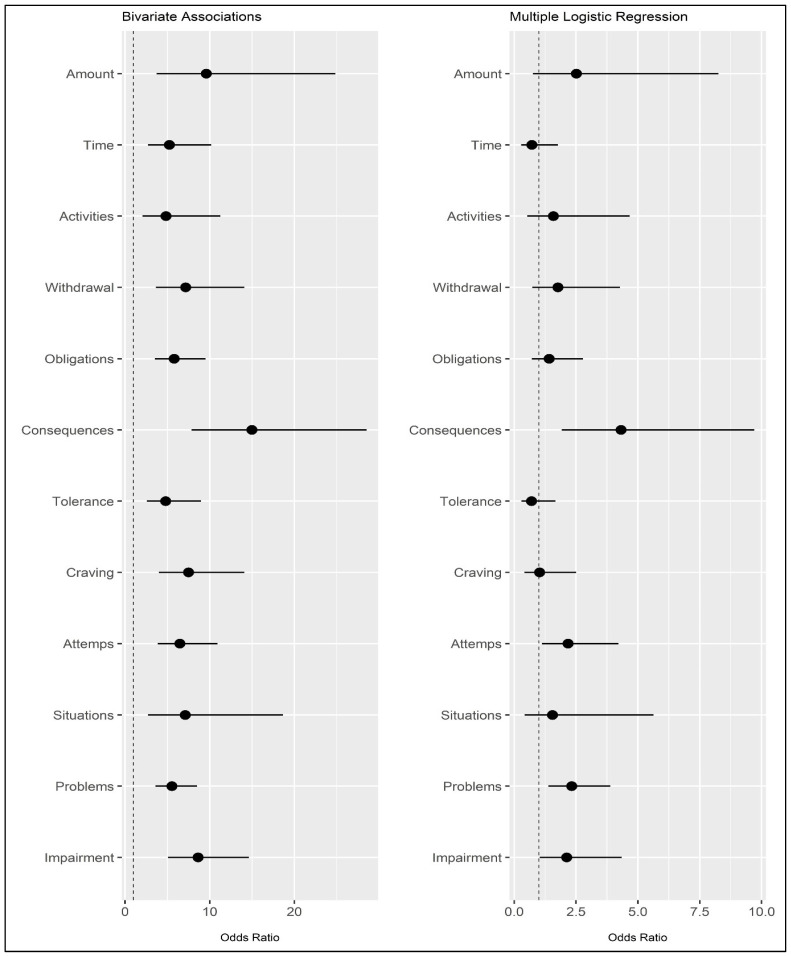
Odds ratios and their confidence intervals (95%) for bivariate associations (**left**) and multiple logistic regression (**right**). *Note*: Dependent variable: grazing (0 = non-problematic vs. 1 = problematic). Independent variable: FA criteria measured with the mYFAS2.0 (0 = absent vs. 1 = present). The dotted line is positioned at the value of 1 (OR not statistically significant).

A binary logistic regression analysis was conducted, regressing the FA criteria (independent variables: 0 = not endorsed vs. 1 = endorsed) onto grazing (dependent variable: 0 = non-problematic grazing vs. 1 = problematic grazing). The model demonstrated a good fit to the data: Hosmer–Lemeshow’s test = 4.134; *p* = 0.530 *ns*. When considering all FA criteria simultaneously, many of the previously observed associations are no longer statistically significant (Table 4 and Figure 4). However, it should be noted that four criteria were still significantly associated with grazing. Specifically, these were Criterion F (“*Use despite knowledge of adverse consequences*”: β = 1.463; OR = 4.319, 95%CI_OR_ [1.922; 9.707]), Criterion I (“*Persistent desire or unsuccessful attempts to quit*”: B = 0.779; OR = 2.180, 95%CI_OR_ [1.126; 4.221]), Criterion K (“*Continued use despite social or interpersonal problems*”: β = 0.844; OR = 2.325, 95%CI_OR_ [1.389, 3.892]), and Criterion L (“*Use causes clinically significant impairment or distress*”: β = 0.755, OR = 2.127, 95%CI_OR_ [1.040, 4.348]). The degree of explained variance (PseudoR^2^) according to Cox and Snell was 0.320, and according to Nagelkerke, it was 0.426. Results are reported in Table 4 and Figure 3.

## 4. Discussion

The global prevalence of obesity is on the rise [2,3], and a contributing factor may be the concept that certain foods can trigger a dependency response, known as FA [10,11,12]. FA is associated with dysfunctional eating behaviors [53], including excessive thoughts about food and the use of food as a regulator of intense emotions [42,43,44,45]—which can lead individuals to experience a loss of control, leading to binge eating behaviors. While binge eating is often associated with FA, recent research suggests that FA is also linked to other problematic eating patterns, such as grazing behavior [55]—the constant, repetitive, compulsive, and unplanned consumption of small quantities of food [55,58].

FA and grazing represent distinct eating behaviors that often intersect and reinforce each other, contributing to unhealthy eating patterns and potential weight-related issues. Grazing on addictive foods can, indeed, reinforce cravings associated with FA, as each instance of grazing provides an opportunity for individuals to consume more of the foods they are addicted to, thus perpetuating the cycle of cravings and consumption. Moreover, grazing can contribute to a loss of control over eating, particularly when individuals continuously consume foods they find addictive. The lack of structured mealtimes and constant access to food can further make it challenging for individuals to regulate their intake and resist cravings. Moreover, the combination of FA and grazing can create a negative reinforcement loop, where individuals consume addictive foods in response to cravings, which in turn reinforces the addictive behaviors and leads to further grazing and overeating. This can exacerbate symptoms of BED, with individuals experiencing intense cravings for specific foods and engaging in frequent episodes of compulsive overeating, and also contribute to weight gain and obesity, as individuals consume excessive calories from addictive foods throughout the day without regard for hunger cues or nutritional balance.

Still, more empirical confirmation is needed to establish the relationship between grazing and FA.

This cross-sectional study had two main objectives. First, (A) aimed to investigate the psychometric properties of the Italian version of the Rep(Eat)-Q, designed to measure grazing in a large sample of patients with severe obesity. Additionally, (B) aimed to examine the association between the presence of problematic grazing and the acceptance of FA criteria in a large sample of patients with severe obesity.

Considering the first aim of the study, the results of the CFA showed that the Rep(Eat)-Q has a first-order two-factor structure with a good fit to the data. Furthermore, all items loaded in a high (λs ≥ 0.69) and statistically significant way on their respective factors, namely repetitive eating and compulsive eating. Moreover, the internal consistency was good for all the grazing scales. In summary, the Rep(Eat)-Q emerges as a short, useful, reliable, and statistically valid instrument for evaluating repetitive eating behaviors, particularly grazing among people with obesity. Its robust psychometric characteristics present notable benefits for both research aims and clinical evaluations.

Furthermore, these findings support the convergent validity of the Rep(Eat)-Q, as it was positively associated in a statistically significant way with measures of uncontrolled eating, binge eating, compulsive eating, and FA. In particular, these findings are consistent with the current scientific literature showing that repetitive eating might be more strongly related to uncontrolled and binge eating behaviors that make individuals more prone to excessive food intake [55,58,94]. On the one hand, these results support evidence showing that repetitive eating could be a potential predictor of binge eating behaviors [55,58,60]. On the other hand, the compulsive grazing scale shows a strong positive association with compulsive eating, suggesting that the grazing behavior may be the result of an urge to eat that cannot be postponed [54,55,58,61]. In the end, all grazing scales (total, RE, CG) are strongly associated with the FA symptom count score.

Furthermore, it is worth emphasizing that the results show how females have (slightly/moderately) higher levels of grazing in all its facets, namely, repetitive eating and compulsive grazing. In particular, it appears that the levels of compulsive grazing are higher (*d* = |0.456|) in this regard, suggesting that females may be (slightly) more prone to the inability to resist feelings and sensations related to food.

Considering the second objective of this study, the frequency analysis (Table 3 and Figure 2) shows that there is a systematic association between the presence of all FA criteria and the presence of problematic grazing. In particular, subjects who meet the FA criteria are those with problematic grazing and vice versa—it should be noted that it is not possible to establish temporal/causal relationships between variables.

Taking into account the bivariate association and not controlling for all independent variables, people with problematic grazing appear to be more prone to consume food despite knowledge of adverse effects (Criterion F; ϕ = 0.480), to eat despite social or interpersonal problems (Criterion K; ϕ = 0.399), to try unsuccessfully try to stop eating (Criterion I; ϕ = 0.374), to not be able to fulfill major role obligations (Criterion E; ϕ = 3.66), and to develop craving symptoms (Criterion H, ϕ = 0345).

Consistent with these results, the logistic regression analysis, controlling for all independent variables, appears to confirm the previously observed results. It suggests that, among all the criteria for food addiction, only four appear to play a statistically significant role in the association with problematic grazing. Specifically, individuals with problematic grazing appear to have a 4.32 times higher risk of eating despite the awareness that such behavior will have negative consequences (Criterion F). Simultaneously, people with problematic grazing would also show a 2.33 times higher risk of encountering social and interpersonal problems (Criterion K); a 2.18 times higher risk of being unable to stop during substance use (Criterion I); and a 2.13 times higher risk of having clinically significant problems or distress (Criterion L).

These results suggest that problematic grazing is not only correlated with aspects intuitively linked to excessive food intake, which can lead the individual to develop obesity. Problematic grazing is also related to aspects and symptoms of FA more closely associated with addiction itself, such as craving, social and interpersonal problems, and the inability to stop using the substance (food). In particular, in this last aspect, the overlap between symptoms of FA and grazing is evident, specifically in the compulsive eating component (grazing) that the individual is unable to interrupt (addiction).

Thus, considering patients with severe obesity, the association between problematic grazing and Criteria I could be particularly important. It is commonly believed that people with FA predominantly engage in binge eating behaviors, consuming excessive amounts of food in a short period [35,53,54]. Consequently, there is a tendency to think that bariatric surgery can prevent the behavioral manifestation of FA, such as loss of control, binge eating, compulsive overeating, and severe obesity [95,96,97,98,99,100]. However, it is not often considered that FA can also manifest as the compulsive eating of small amounts of food over a long (but consistent) period—namely, compulsive grazing [49,55]. Supporting this hypothesis, the literature has shown that individuals with problematic grazing undergoing bariatric surgery appear to be more prone to failing in maintaining weight lost immediately after surgery, suggesting that the absence/presence of grazing could be a central predictor of the success/failure of these interventions [49,60,62,63]. Therefore, the demonstrated association suggests that FA can also manifest through problematic grazing. Therefore, those who show problematic grazing have the typical behavioral profile of subjects with FA.

### 4.1. Limitations and Strengths

Regarding the limitations of this study, the cross-sectional research design did not allow for the testing of the stability of the results over time; the sample consisted only of patients with obesity, thus limiting the generalizability of results to other populations; and only self-report assessment tools were used despite their potential proneness to social desirability biases. Furthermore, factors such as dieting practices, medication use, and socioeconomic status were not considered. Future research may try to overcome these limitations by extending this study to other populations (e.g., community samples) and by using a longitudinal study design monitoring the levels of psychological variables over time [101]. This will allow for the testing of the measurement invariance of the Rep(Eat)-Q across various countries and longitudinally. Future studies will also test the discriminant validity of the Rep(Eat)-Q with other measures related to eating [102].

Regarding the strengths of this research, this is the first Italian study that provides the validation of the Rep(Eat)-Q which is widely used as it can predict the failure of bariatric surgery in weight reduction [49,60,62,63]. If regularly included in the assessment batteries, the Rep(Eat)-Q can lead to several benefits, which are both clinical and economic. The other strengths of this research are the wide sample that allowed accurate estimates in the statistical models, the good psychometric properties of the tool, and the use of rigorous and well-established statistical methods according to current guidelines. Furthermore, the demonstrated associations seem to suggest that FA may manifest not only through loss of control and excessive food intake but also through compulsive and constant behaviors. This implies alternative pathways for conceptualizing the constructs of grazing and FA.

### 4.2. Clinical Implications

The Rep(Eat)-Q and the results previously showed have crucial clinical implications. Providing validation of a questionnaire measuring grazing and showing its significant association with FA can raise awareness about the importance of these constructs, grazing, and FA among healthcare professionals.

Indeed, the absence of specific criteria for diagnosing FA and grazing can make it challenging for clinicians to identify these behaviors within the framework of traditional psychiatric diagnoses. Professionals, therefore, need to rely on measures of established validity and reliability to identify and address these behaviors and the underlying contributors.

Furthermore, considering that many people with obesity undergo bariatric surgery to achieve weight reduction, the majority experience weight gain after an initial decrease, with the inability to consume large amounts of food quickly leading them to adopt grazing as an eating strategy, which is potentially influenced by an underlying FA. Thus, these findings show that grazing is strongly associated with FA criteria, suggesting that individuals exhibiting problematic grazing demonstrate a typical behavioral profile of subjects with FA. Thus, targeting grazing may indirectly lower FA levels, consequently helping patients with obesity to recover functional eating behaviors.

## 5. Conclusions

In conclusion, evaluating grazing habits emerges as a worthwhile strategy to improve weight management in individuals with severe obesity. Identifying this problematic eating pattern can pose challenges due to its minimal psychological impact, which is often overlooked by both patients and clinicians. However, adopting the Rep(Eat)-Q, a brief self-report questionnaire with good psychometric properties, offers valuable clinical insights to inform practitioners’ efforts.

## Figures and Tables

**Table 1:** Item descriptive statistics, item psychometric properties, and confirmation factor analysis.

	Descriptive				Factor	Properties	Confirmatory Factor Analysis	
	Mean	SD	SK	K		*r*_(it-tot)_	λ	*R* ^2^
Item#1	1.06	1.59	1.54	1.41	RE	0.667	0.689	0.475
Item#2	1.70	1.75	0.82	−0.41	RE	0.840	0.857	0.735
Item#3	1.82	1.90	0.81	−0.58	RE	0.819	0.841	0.708
Item#4	1.54	1.80	1.04	−0.07	RE	0.868	0.886	0.785
Item#9	1.87	1.86	0.66	−0.81	RE	0.776	0.836	0.700
Item#10	1.82	1.84	0.74	−0.65	RE	0.855	0.899	0.808
Item#5	1.39	1.75	1.12	0.04	CG	0.767	0.799	0.638
Item#6	1.73	1.79	0.82	−0.51	CG	0.818	0.874	0.764
Item#7	1.91	1.87	0.65	−0.82	CG	0.840	0.892	0.796
Item#8	1.70	1.85	0.93	−0.08	CG	0.795	0.821	0.674
Item#11	1.72	1.98	0.90	−0.48	CG	0.701	0.718	0.515
Item#12	2.28	2.05	0.46	−1.08	CG	0.803	0.834	0.695

*Note*: SD = standard deviation, SK = skewness, K = kurtosis; *r*_(it-tot)_ = adjusted item-total correlation of the specific factor; λ = standardized factor loading; *R*^2^ = explained variance.

**Table 2:** Correlations among variables.

		Descriptive		Correlations								
		M	SD	1	2	3	4	5	6	7	8	9
1	Rep(Eat)-Q	1.71	1.53	-								
2	Rep(Eat)-Q—RE	1.64	1.56	0.965 **	-							
3	Rep(Eat)-Q—CG	1.79	1.61	0.967 **	0.867 **	-						
4	mYFAS2.0	2.43	2.67	0.694 **	0.639 **	0.701 **	-					
5	BES	11.42	8.93	0.694 **	0.633 **	0.706 **	0.696 **	-				
6	MEC10	14.74	10.34	0.705 **	0.623 **	0.737 **	0.688 **	0.773 **	-			
7	TFEQ-R-18—CR	13.64	3.48	−0.079	−0.099	−0.056	−0.076	−0.143	−0.129	-		
8	TFEQ-R-18—UE	17.61	6.66	0.753 **	0.690 **	0.762 **	0.667 **	0.728 **	0.823 **	−0.079	-	
9	TFEQ-R-18—EE	7.07	3.05	0.643 **	0.553 **	0.684 **	0.573 **	0.585 **	0.648 **	−0.003	0.670 **	-
10	BMI	42.28	6.48	−0.011	−0.011	−0.010	0.061	0.026	0.037	−0.156 *	0.000	−0.048

*Note*: * = *p* < 0.050; ** = *p* < 0.001. Rep(Eat)-Q = total score; Rep(Eat)-Q—RE = repetitive eating; Rep(Eat)-Q—CG = compulsive grazing; mYFAS2.0 = modified Yale Food Addiction Scale 2.0 symptom count; BES = Binge Eating Scale; MEC10 = Measure of Eating Compulsivity 10; TFEQ-R-18—CR = cognitive restraint; TFEQ-R-18—UE = uncontrolled eating; TFEQ-R-18—EE = emotional eating; BMI = body mass index.

**Table 3:** Contingency tables.

			Grazing		χ^2^	Phi	OR
			No	Yes		(ϕ)	
Criterion A	Larger amount and for a longer period than intended	No	192	5	30.229	0.274	9.600
		Yes	164	41			
Criterion B	Much time/activity to obtain, use, recover	No	185	12	27.881	0.263	5.239
		Yes	153	52			
Criterion C	Social […] activities given up or reduced	No	190	7	15.708	0.198	4.836
		Yes	174	31			
Criterion D	Characteristic withdrawal symptoms	No	186	11	39.924	0.315	7.163
		Yes	144	61			
Criterion E	Failure to fulfill major role obligation	No	171	26	53.755	0.366	5.792
		Yes	109	96			
Criterion F	[…] Despite knowledge of adverse consequences	No	185	12	92.677	0.480	14.972
		Yes	104	101			
Criterion G	Tolerance	No	183	14	27.484	0.261	4.793
		Yes	150	55			
Criterion H	Craving	No	184	13	47.769	0.345	7.499
		Yes	134	71			
Criterion I	Persistent desire or unsuccessful attempts to quit	No	175	22	56.193	0.374	6.476
		Yes	113	92			
Criterion J	Use in physically hazardous situations	No	192	5	20.541	0.226	7.103
		Yes	173	32			
Criterion K	Social or interpersonal problems	No	125	72	64.009	0.399	5.527
		Yes	49	156			
Criterion L	Clinically significant impairment or distress	No	176	21	75.289	0.433	8.630
		Yes	101	104			

*Note*: each χ^2^ analysis has 1 *df*; Phi = effect size; OR = odds ratio for the simple bivariate association. Criterion A = “Substance taken in larger amount and for a longer period than intended”; Criterion B = “Much time/activity to obtain, use, recover”; Criterion C = “Important social, occupational, or recreational activities given up or reduced”; Criterion D = “Characteristic withdrawal symptoms; substance taken to relieve withdrawal”; Criterion E = “Failure to fulfill major role obligation (e.g., work, school, home)”; Criterion F = “Use continues despite knowledge of adverse consequences (e.g., emotional problems, physical problems)”; Criterion G = “Tolerance (marked increase in amount; marked decrease in effect)”; Criterion H = “Craving, or a strong desire or urge to use”; Criterion I = “Persistent desire or repeated unsuccessful attempts to quit”; Criterion J = “Use in physically hazardous situations”; Criterion K = “Continued use despite social or interpersonal problems”; Criterion L “Use causes clinically significant impairment or distress”.

**Table 4:** Logistic regression analysis.

Criterion	Symptom	β (se)	*z*-Value	*p*-Value	OR	OR 95% [L; U]
Criterion A	Amount	0.922 (0.606)	1.522	0.128	2.515	[0.767; 8.253]
Criterion B	Time	−0.332 (0.463)	−0.718	0.473	0.717	[0.290; 1.776]
Criterion C	Activities	0.461 (0.552)	0.836	0.403	1.586	[0.538; 4.677]
Criterion D	Withdrawal	0.571 (0.450)	1.270	0.204	1.771	[0.733; 4.278]
Criterion E	Obligations	0.348 (0.344)	1.010	0.312	1.416	[0.721; 2.780]
Criterion F	Consequences	1.463 (0.413)	3.541	<0.001 ***	4.319	[1.922; 9.707]
Criterion G	Tolerance	−0.356 (0.442)	−0.805	0.421	0.700	[0.294; 1.667]
Criterion H	Craving	0.031 (0.455)	0.067	0.946	1.031	[0.423; 2.513]
Criterion I	Attempts	0.779 (0.337)	2.312	0.021 *	2.180	[1.126; 4.221]
Criterion J	Situations	0.436 (0.659)	0.662	0.508	1.546	[0.425; 5.629]
Criterion K	Problems	0.844 (0.263)	3.211	0.001 **	2.325	[1.389; 3.892]
Criterion L	Impairment	0.755 (0.365)	2.068	0.039 *	2.127	[1.040; 4.348]

*Note*: * = *p* < 0.050; ** = *p* < 0.010; *** = *p* < 0.001. β = unstandardized logistic regression coefficient; se = standard error; OR = odds ratio; 95% [L; U] = 95% confidence interval for the odds ratio. Criterion A = “Substance taken in larger amount and for a longer period than intended”; Criterion B = “Much time/activity to obtain, use, recover”; Criterion C = “Important social, occupational, or recreational activities given up or reduced”; Criterion D = “Characteristic withdrawal symptoms; substance taken to relieve withdrawal”; Criterion E = “Failure to fulfill major role obligation (e.g., work, school, home)”; Criterion F = “Use continues despite knowledge of adverse consequences (e.g., emotional problems, physical problems)”; Criterion G = “Tolerance (marked increase in amount; marked decrease in effect)”; Criterion H = “Craving, or a strong desire or urge to use”; Criterion I = “Persistent desire or repeated unsuccessful attempts to quit”; Criterion J = “Use in physically hazardous situations”; Criterion K = “Continued use despite social or interpersonal problems”; Criterion L “Use causes clinically significant impairment or distress”.

## Data Availability

Data are available on a reasonable request due to privacy and ethical restrictions.

## Supplementary Materials

The following supporting information can be downloaded at https://www.mdpi.com/article/10.3390/nu16070949/s1; File S1: The Italian version of the Rep(Eat)-Q.
