# Supplementary material for: The Association between Grazing and Food Addiction: The Italian Version of the Repetitive Eating Questionnaire (Rep(Eat)-Q) and Its Relationships with Food Addiction Criteria
Source: Nutrients. 2024 Mar 26;16(7):949. doi: 10.3390/nu16070949 (PMC11013059; doi:10.3390/nu16070949)
Supplement: Supplementary file 1 [file nutrients-16-00949-s001.zip › nutrients-2911172-supplementary.pdf]

Repetitive Eating Questionnaire (Rep(Eat)-Q)

ISTRUZIONI:

La preghiamo di **rispondere a tutte le domande** ponendo un **segno in corrispondenza del valore numerico** – compreso tra **0 (MAI)** e **6 (OGNI GIORNO)** – **che meglio rappresenta quanto spesso**, ***nelle ultime 4 settimane***, **ha agito i seguenti comportamenti o abitudini**.

Il termine **"SPILUCCARE"** indica: **mangiare** ripetutamente **piccole** – o **modeste** – **quantità di cibo**, al di **fuori dei pasti previsti/programmati** dal proprio regime dietetico.

0	1	2	3	4	5	6
MAI	RARAMENTE	OCCASIONALMENTE	POCHI GIORNI	MOLTI GIORNI	LA MAGGIOR PATE DEI GIORNI	OGNI GIORNO

***Nelle ultime 4 settimane*:**

**1**	Ha mangiato tutto il giorno **in maniera intermittente**, **senza averlo programmato**.	**0**	**1**	**2**	**3**	**4**	**5**	**6**
**2**	Ha mangiato **ripetutamente** piccole o modeste quantità di cibo tra un pasto e l'altro, **senza averlo programmato prima**.	**0**	**1**	**2**	**3**	**4**	**5**	**6**
**3**	Ha spiluccato **senza badare a ciò che stava facendo**.	**0**	**1**	**2**	**3**	**4**	**5**	**6**
**4**	Ha spiluccato **ripetutamente, durante tutto il giorno,** tra un pasto e l'altro.	**0**	**1**	**2**	**3**	**4**	**5**	**6**
**5**	Non voleva mangiare, ma sentiva che **non poteva evitare di farlo**.	**0**	**1**	**2**	**3**	**4**	**5**	**6**
**6**	**Non è riuscito/a a resistere** dallo spiluccare anche se aveva provato a non farlo.	**0**	**1**	**2**	**3**	**4**	**5**	**6**
**7**	**Ha fatto fatica a controllare il desiderio** di mangiare tra un pasto e l'altro.	**0**	**1**	**2**	**3**	**4**	**5**	**6**
**8**	**Si è sentito/a spinto** a mangiare.	**0**	**1**	**2**	**3**	**4**	**5**	**6**
**9**	Ha spiluccato **senza pensare a quanto stava mangiando**.	**0**	**1**	**2**	**3**	**4**	**5**	**6**
**10**	Ha mangiato **senza averlo programmato tra i pasti** e gli spuntini stabiliti.	**0**	**1**	**2**	**3**	**4**	**5**	**6**
**11**	**Si è sentito/a a turbato** dopo aver spiluccato.	**0**	**1**	**2**	**3**	**4**	**5**	**6**
**12**	Ha spiluccato **quando era ansioso/a, annoiato/a, solo/a oppure** quando **provava altre emozioni**.	**0**	**1**	**2**	**3**	**4**	**5**	**6**
